# Cefiderocol nonsusceptibility among carbapenem-resistant *Pseudomonas aeruginosa* and *Acinetobacter baumannii*: data from the 2024 Antimicrobial Testing Leadership and Surveillance (ATLAS) program

**DOI:** 10.1128/aac.00634-26

**Published:** 2026-07-13

**Authors:** Po-Wei Chiu, Jiun-Ling Wang, Shun-Chung Hsueh, Nan-Yao Lee, Yu-Tsung Huang, Wen-Chien Ko, Po-Ren Hsueh

**Affiliations:** 1 Department of Internal Medicine, National Cheng Kung University Hospital 639603, Tainan, Taiwan; 2 Department of Medicine, College of Medicine, National Cheng Kung University 665116, Tainan, Taiwan; 3 Division of Pulmonary and Critical Care Medicine, Department of Internal Medicine, National Taiwan University Hospital 568619, Taipei City, Taiwan; 4 Center for Infection Control, National Cheng Kung University Hospital, College of Medicine, National Cheng Kung University 535218, Tainan, Taiwan; 5 Department of Laboratory Medicine, National Taiwan University Hospital, National Taiwan University College of Medicine 607281, Taipei City, Taiwan; 6 Division of Infectious Diseases, Department of Internal Medicine, China Medical University Hospital, School of Medicine, China Medical University 38020, Taichung, Taiwan; 7 Department of Laboratory Medicine, China Medical University Hospital, School of Medicine, China Medical University 38020, Taichung, Taiwan; Entasis, Big Bay, Michigan, USA

**Keywords:** cefiderocol nonsusceptibility, carbapenem-resistant *Pseudomonas aeruginosa*, carbapenem-resistant *Acinetobacter baumannii*, metallo-β-lactamase, Antimicrobial Testing Leadership and Surveillance program

## Abstract

We evaluated cefiderocol activity against carbapenem-resistant *Pseudomonas aeruginosa* (CRPA) and carbapenem-resistant *Acinetobacter baumannii* (CRAB), characterized the β-lactamase gene profiles of cefiderocol-nonsusceptible CRPA by metallo-β-lactamase (MBL) subtype, and assessed cross-resistance with comparator agents. Cefiderocol minimum inhibitory concentrations (MICs) for 890 CRPA and 1,063 CRAB isolates from the 2024 Antimicrobial Testing Leadership and Surveillance (ATLAS) program were determined by broth microdilution using iron-depleted cation-adjusted Mueller-Hinton broth. Cefiderocol nonsusceptibility was defined as MIC ≥ 8 mg/L per CLSI (intermediate + resistant). DTRPA was defined as nonsusceptible to all anti-pseudomonal β-lactams and fluoroquinolones; DTRAB was defined as nonsusceptible to carbapenems, ampicillin-sulbactam, and fluoroquinolones, reflecting species-specific susceptibility panels. CRPA isolates underwent molecular screening for *bla*_NDM_, *bla*_VIM_, *bla*_IMP_, serine carbapenemase, and ESBL genes; genotype data were not available for *A. baumannii*. Cefiderocol susceptibility was 93.9% for CRPA and 88.5% for CRAB, respectively (nonsusceptibility 6.1% and 11.5%, respectively; OR = 2.01, *P* < 0.001); nonsusceptibility among DTRPA and DTRAB was 8.1% and 11.7%, respectively. Among 54 cefiderocol-nonsusceptible CRPA, 41 (76%) harbored no detectable carbapenemase gene. Nonsusceptibility among MBL-positive CRPA was exclusively attributable to *bla*_NDM_ producers (27.7%, 13/47); *bla*_VIM_-positive (*n* = 68) and *bla*_IMP_-positive (*n* = 21) isolates were uniformly susceptible. Among cefiderocol-nonsusceptible CRPA, 98.1% were co-resistant to ceftazidime-avibactam and 96.3% to ceftolozane-tazobactam.

## INTRODUCTION

Carbapenem-resistant *Pseudomonas aeruginosa* (CRPA) and carbapenem-resistant *Acinetobacter baumannii* (CRAB) are classified as critical priority pathogens by the World Health Organization (1). Infections caused by these organisms are associated with high mortality and limited therapeutic options (2). Difficult-to-treat resistance (DTR), defined by nonsusceptibility to all first-line β-lactams and fluoroquinolones (3), identifies isolates with the most constrained treatment options and poses a particular clinical challenge. CRAB exhibits intrinsically high rates of multidrug resistance across geographic regions (2). Ceftazidime-avibactam and ceftolozane-tazobactam have expanded the treatment options for CRPA, but neither possesses activity against metallo-β-lactamase (MBL)-producing strains (4). For CRAB, sulbactam-durlobactam in combination with a carbapenem is recommended (4). Cefiderocol is a siderophore cephalosporin with stability against all four Ambler classes of β-lactamases (5, 6). Randomized trials have demonstrated its efficacy for serious gram-negative infections (7, 8), and current guidelines recommend cefiderocol as the preferred agent for MBL-producing DTR *P. aeruginosa* (4, 9, 10). Infectious Diseases Society of America (IDSA) reserves cefiderocol as a last-resort option in CRAB due to testing challenges, signals of inferior efficacy in CREDIBLE-CR, and limited observational data (4, 8).

Critical knowledge gaps remain. First, previous surveillance data were geographically concentrated in North America and Europe (11, 12). The updated SENTRY data (2020–2024) have demonstrated sustained cefiderocol activity overall, but region-specific analysis for carbapenem-resistant subsets remains limited (13, 14). Regions with the highest MBL burden, notably South Asia, remain underrepresented (2). A recent meta-analysis reported an overall cefiderocol nonsusceptibility of 1.4% for *P. aeruginosa* and 8.8% for *A. baumannii*, applying EUCAST PK/PD (non-species-related) breakpoints (susceptible ≤2 mg/L, resistant >2 mg/L) to *A. baumannii* in the absence of species-specific clinical breakpoints (15). Second, in the setting of DTR *P. aeruginosa* and DTR *A. baumannii*, the therapeutic options are limited, and the clinical efficacy of available agents remains uncertain. Comprehensive *in vitro* data on newer antimicrobial agents, including cefiderocol and aztreonam-avibactam, are critically needed to define the current resistance landscape and guide empirical therapy. Third, cross-resistance profiles between cefiderocol and comparator agents stratified by genotype have not been evaluated.

This study leveraged the Antimicrobial Testing Leadership and Surveillance (ATLAS) program (2, 16) to address these gaps, evaluating cefiderocol activity against CRPA and CRAB isolates collected globally in 2024 with three objectives: (i) characterize nonsusceptibility rates and geographic distribution; (ii) delineate β-lactamase gene profiles of cefiderocol-nonsusceptible CRPA, with emphasis on MBL subtype-specific activity; and (iii) assess cross-resistance profiles across genotype groups.

## RESULTS

### Bacterial isolates

In 2024, the ATLAS program collected 4,845 *P. aeruginosa* and 1,516 *A. baumannii* isolates globally. Of these, 890 (18.4%) *P*. *aeruginosa* met the definition for CRPA, and 1,063 (70.1%) *A*. *baumannii* met the definition for CRAB. Independently, 643 *P. aeruginosa* (13.3% of all *P. aeruginosa*) and 1,042 *A. baumannii* (68.7% of all *A. baumannii*) met the difficult-to-treat resistance criteria of Kadri et al. (3) for DTRPA and DTRAB, respectively. Consistent with the inherently high resistance burden in *A. baumannii*, a substantially greater proportion of *A. baumannii* than *P. aeruginosa* qualified as DTR (68.7% vs 13.3%). By continent, Europe contributed the most CRPA isolates (*n* = 386, 43.4%), followed by Asia (*n* = 256, 28.8%), Latin America (*n* = 164, 18.4%), and Africa/Middle East (*n* = 53, 6.0%). For CRAB, Europe also contributed the most isolates (*n* = 418, 39.3%), followed by Asia (*n* = 348, 32.7%), Latin America (*n* = 155, 14.6%), and Africa/Middle East (*n* = 122, 11.5%). By region, the proportion of *P. aeruginosa* meeting CRPA criteria was 23.3% in Asia (256/1,099), 22.4% in Latin America (164/731), 16.9% in Europe (386/2,289), 12.9% in Africa/Middle East (53/410), 8.6% in North America (13/152), and 5.5% in Oceania (9/164); for *A. baumannii*, the proportion meeting CRAB criteria was 81.3% in Asia (348/428), 67.4% in Africa/Middle East (122/181), 67.1% in Europe (418/623), 64.9% in Latin America (155/239), 13.3% in North America (2/15), and 6.7% in Oceania (2/30).

### *In vitro* activity of cefiderocol

The *in vitro* activity of cefiderocol, stratified by resistance phenotype, is summarized in Table 1. Among CRPA, susceptibility was 93.9% (836/890), with nonsusceptibility of 6.1% (54/890; intermediate 4.5% and resistant 1.6%); minimum inhibitory concentration (MIC_50_/MIC_90_) was 0.5/4 mg/L. Among DTRPA, nonsusceptibility increased modestly to 8.1% (52/643).

**TABLE 1:** *In vitro* activity of cefiderocol against carbapenem-resistant and difficult-to-treat resistant *P. aeruginosa* and *A. baumannii^a^*

Population	No. of isolates	CLSI-S (%)	CLSI-I (%)	CLSI-R (%)	EUCAST-S (%)	EUCAST-R (%)	MIC_50_	MIC_90_
*P. aeruginosa*								
CRPA^*b*^	890	93.9	4.5	1.6	88.4	11.6	0.5	4
DTRPA^*c*^	643	91.9	6.1	2.0	84.6	15.4	0.5	4
*A. baumannii*								
CRAB	1,063	88.5	4.6	6.9	IE	IE	0.5	8
DTRAB	1,042	88.3	4.7	7.0	IE	IE	0.5	8

^*a*^ CLSI cefiderocol breakpoints (M100, 2024): *S* ≤ 4, *I* = 8, *R* ≥ 16 mg/L. Nonsusceptibility = *I* + *R* (MIC ≥ 8 mg/L). EUCAST cefiderocol breakpoints (v14.0, 2024): *S* ≤ 2, *R* > 2 mg/L (*P. aeruginosa* only). IE = insufficient evidence (*A. baumannii*).

^*b*^ CRPA/CRAB defined as meropenem-resistant (MIC ≥ 8 mg/L, CLSI).

^*c*^ DTRPA/DTRAB defined per Kadri et al. (3). DTR was applied to the full species population independently of CR; the two populations overlap but are not nested.

Among CRAB, susceptibility was 88.5% (941/1,063), with nonsusceptibility of 11.5% (122/1,063; intermediate 4.6% and resistant 6.9%); MIC_50_/MIC_90_ was 0.5/8 mg/L. Among DTRAB, nonsusceptibility was 11.7% (122/1,042). CRAB had a significantly higher cefiderocol nonsusceptibility rate than CRPA (11.5% vs 6.1%; odds ratio [OR] = 2.01, *P* < 0.001). EUCAST breakpoints yielded higher nonsusceptibility rates for *P. aeruginosa*, as reported in the Table 1 footnote.

### Nonsusceptibility across resistance mechanisms in CRPA

**Fig 1:**
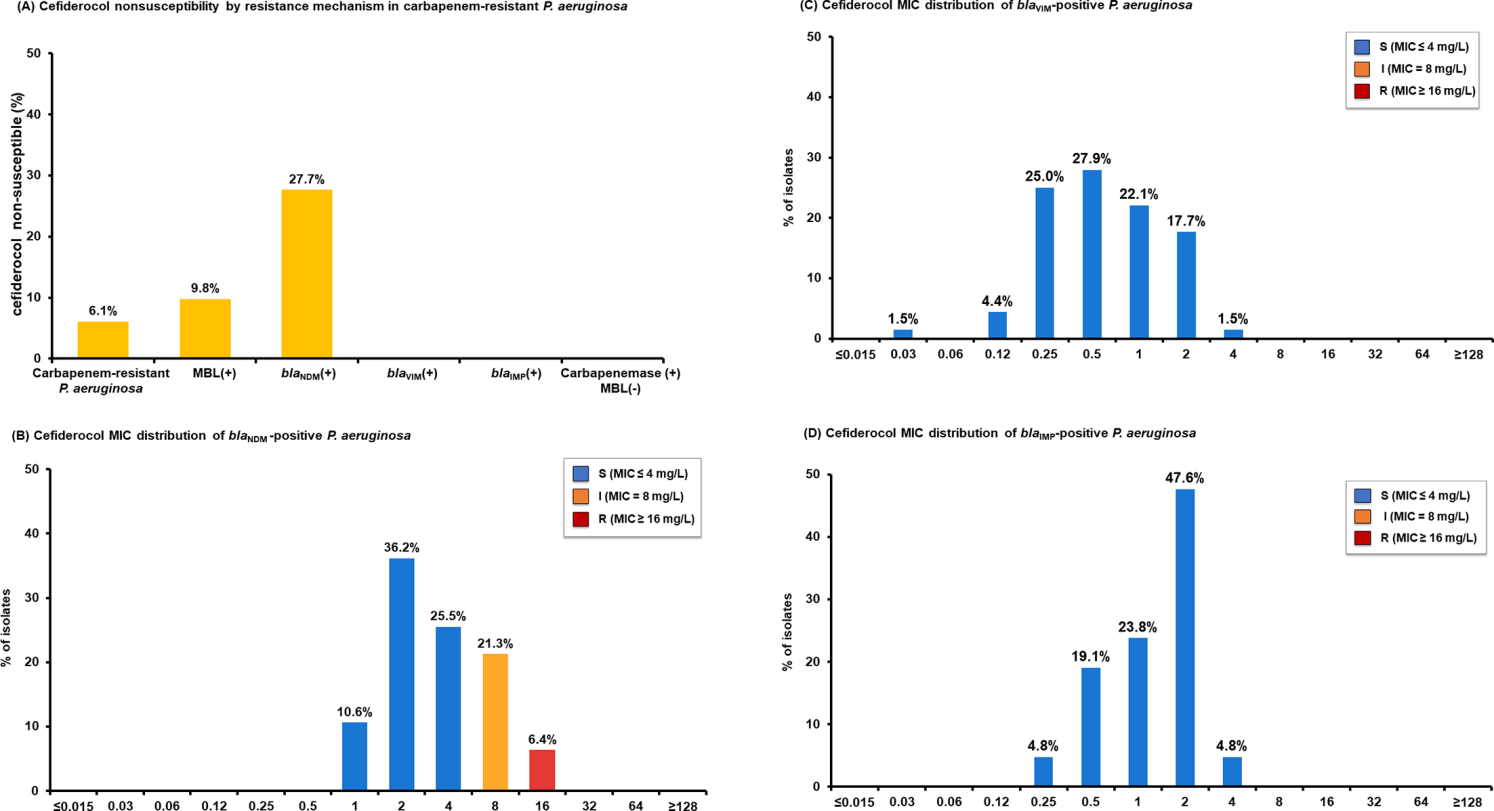
Cefiderocol activity stratified by β-lactamase mechanism among carbapenem-resistant *P. aeruginosa*. (**A**) Cefiderocol nonsusceptibility cascade across resistance mechanism categories: CRPA overall (*n* = 890), MBL-positive (*n* = 133), *bla*_NDM_-positive (*n* = 47), *bla*_VIM_-positive (*n* = 68), *bla*_IMP_-positive (*n* = 21), and non-MBL carbapenemase (carbapenemase-positive/MBL-negative; *n* = 25). MBL-positive includes carriage of any *bla*_NDM_, *bla*_VIM_, or *bla*_IMP_. Non-MBL carbapenemase isolates carried *bla*_KPC_ or *bla*_GES_ without co-carriage of any MBL gene. Categories are not mutually exclusive because three isolates co-harbored more than one MBL gene. (**B–D**) Cefiderocol MIC distributions for *bla*_NDM_-positive (**B**), *bla*_VIM_-positive (**C**), and *bla*_IMP_-positive (**D**) *P. aeruginosa*. Percentages on the *y*-axis reflect the proportion of isolates within each MBL-positive subset.

Cefiderocol nonsusceptibility across resistance-mechanism categories is shown in Fig. 1A. MBL-positive CRPA had 9.8% nonsusceptibility (13/133), while carbapenemase-positive/MBL-negative CRPA (*n* = 25) had 0%. Non-carbapenemase-producing CRPA (*n* = 732) had 5.6% nonsusceptibility, accounting for 76% (41/54) of all cefiderocol-nonsusceptible isolates. Among MBL-positive CRPA, nonsusceptibility was exclusively attributable to *bla*_NDM_-producing isolates (27.7%, 13/47); *bla*_VIM_-positive (*n* = 68) and *bla*_IMP_-positive (*n* = 21) isolates were uniformly susceptible (Fig. 1B through D). All Class A β-lactamase carriers (*bla*_GES_, *bla*_KPC_, *bla*_PER_, *bla*_VEB_, *bla*_CTX-M_, and *bla*_TEM_) had 0% nonsusceptibility (Table S1). Among ceftazidime-avibactam-resistant CRPA (*n* = 410), cefiderocol nonsusceptibility was 12.9%, and among ceftolozane-tazobactam-resistant CRPA (*n* = 386), it was 13.5%.

### Geographic distribution of cefiderocol nonsusceptibility

**Fig 2:**
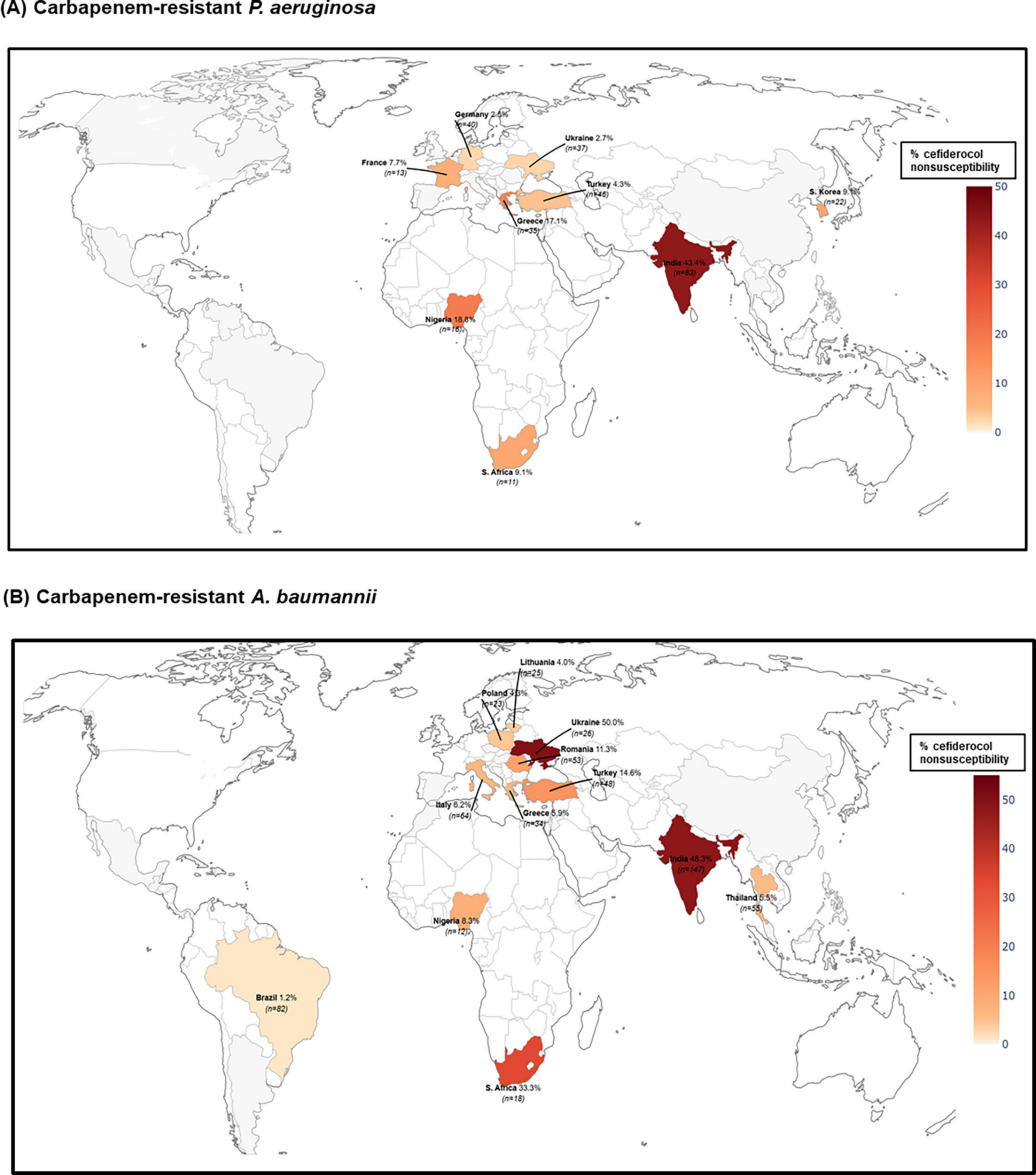
Geographic distribution of cefiderocol nonsusceptibility, ATLAS 2024. Choropleth maps showing country-level cefiderocol nonsusceptibility for (**A**) carbapenem-resistant *P. aeruginosa* and (**B**) carbapenem-resistant *A. baumannii*. Countries contributing fewer than 10 isolates were excluded from the geographic analysis and are shown in gray. Color scale: 0% (light) to 50% nonsusceptibility (deep). The United States was not represented in the 2024 ATLAS data set.

The geographic distribution of cefiderocol nonsusceptibility is shown in Fig. 2. For CRPA (Fig. 2A), among countries contributing ≥10 isolates, the highest nonsusceptibility rate was observed in India (43.4%, 36/83), followed by Nigeria (18.8%, 3/16), Greece (17.1%, 6/35), South Korea (9.1%, 2/22), and South Africa (9.1%, 1/11). By contrast, Turkey (4.3%), Ukraine (2.7%), and Germany (2.5%) had low CRPA nonsusceptibility rates. Several countries in Latin America, North America, and Oceania had 0% nonsusceptibility among CRPA.

For CRAB (Fig. 2B), the geographic distribution was more dispersed. Ukraine had the highest nonsusceptibility rate (50.0%, 13/26), followed by India (48.3%, 71/147), South Africa (33.3%, 6/18), Turkey (14.6%, 7/48), and Romania (11.3%, 6/53). In contrast to CRPA, several European and Asian countries had moderate CRAB nonsusceptibility rates.

By continent, Asia had the highest cefiderocol nonsusceptibility rate for both CRPA (14.8%, 38/256) and CRAB (21.3%, 74/348), respectively (Table 2). Europe had intermediate rates (CRPA 2.8% and CRAB 8.6%, respectively), while Latin America had the lowest rates (CRPA 0% and CRAB 0.6%, respectively).

**TABLE 2:** Clinical characteristics associated with cefiderocol nonsusceptibility among CRPA and CRAB^*a*^

Variable	*N*	FDC-NS (*n*)	FDC-NS (%)	OR (95% CI)	*P*
*P. aeruginosa* (CRPA)					
Continent					
Europe	386	11	2.8	Ref	
Africa/ME	53	5	9.4	3.55 (1.28–10.68)	0.033
Asia	256	38	14.8	5.94 (2.92–11.35)	<0.001
North America	13	0	0.0	0.00 (0.07–21.61)	1.000
Latin America	164	0	0.0	0.00 (0.01–1.69)	0.039
Oceania	9	0	0.0	0.00 (0.09–31.35)	1.000
Specimen source					
Blood	131	10	7.6	Ref	
Respiratory	431	18	4.2	0.53 (0.24–1.13)	0.114
Intra-abdominal	62	5	8.1	1.06 (0.38–3.25)	1.000
Urinary	109	15	13.8	1.93 (0.83–4.34)	0.140
Ward					
General medical	269	8	3.0	Ref	
Medical ICU	201	22	10.9	4.01 (1.71–8.68)	<0.001
General surgical	128	4	3.1	1.05 (0.35–3.56)	1.000
Surgical ICU	71	5	7.0	2.47 (0.84–7.69)	0.156
Age group					
≥61	441	20	4.5	Ref	
0–17	72	5	6.9	1.57 (0.63–4.44)	0.375
18–30	50	4	8.0	1.83 (0.69–5.77)	0.291
31–60	318	25	7.9	1.80 (0.98–3.26)	0.062
*A. baumannii (CRAB*)					
Continent					
Europe	418	36	8.6	Ref	
Africa/ME	122	9	7.4	0.85 (0.42–1.85)	0.852
Asia	348	74	21.3	2.87 (1.86–4.35)	<0.001
North America	2	0	0.0	0.00 (0.10–44.49)	1.000
Latin America	155	1	0.6	0.07 (0.02–0.53)	<0.001
Oceania	2	1	50.0	10.61 (1.06–103.33)	0.169
Specimen source					
Blood	246	29	11.8	Ref	
Respiratory	477	66	13.8	1.20 (0.75–1.89)	0.487
Intra-abdominal	55	4	7.3	0.59 (0.23–1.82)	0.474
Urinary	76	6	7.9	0.64 (0.28–1.66)	0.405
Ward					
General medical	321	22	6.9	Ref	
Medical ICU	272	38	14.0	2.21 (1.26–3.78)	0.006
General surgical	122	13	10.7	1.62 (0.81–3.34)	0.235
Surgical ICU	100	17	17.0	2.78 (1.43–5.45)	0.005
Age group					
≥61	596	53	8.9	Ref	
0–17	70	22	31.4	4.70 (2.66–8.36)	<0.001
18–30	56	6	10.7	1.23 (0.55–3.10)	0.626
31–60	325	40	12.3	1.44 (0.93–2.22)	0.109

^*a*^ FDC-NS: cefiderocol nonsusceptible (CLSI I + R, MIC ≥ 8 mg/L). OR: odds ratio vs pre-specified reference group (ref). Fisher’s exact test; *P* < 0.05.

### Clinical characteristics associated with cefiderocol nonsusceptibility

The association of cefiderocol nonsusceptibility with clinical and epidemiological variables is shown in Table 2, with ORs and *P* values for each stratum. For CRPA, the medical intensive care unit had the highest nonsusceptibility rate (10.9%, 22/201), significantly higher than general medical wards (3.0%, 8/269). By specimen source, nonsusceptibility was numerically higher among urinary (13.8%) and intra-abdominal (8.1%) than respiratory (4.2%) isolates, although none of these differences reached statistical significance (Table 2).

For CRAB, the pediatric age group (0–17 years) had a notably higher nonsusceptibility rate (31.4%, 22/70) than adults aged ≥61 years (8.9%, 53/596), although this finding should be interpreted with caution given the limited sample size.

### β-Lactamase gene distribution among cefiderocol-nonsusceptible CRPA

Among the 54 cefiderocol-nonsusceptible isolates, 13 (24.1%) were MBL-positive and 41 (75.9%) harbored no identifiable carbapenemase gene (Table S1). All 13 MBL-positive cefiderocol-nonsusceptible isolates carried *bla*_NDM_; no cefiderocol-nonsusceptible isolates were identified among *bla*_VIM_ or *bla*_IMP_ carriers. Among MBL-negative cefiderocol-nonsusceptible isolates, none carried *bla*_KPC_, *bla*_GES_, or any tested ESBL gene. Genotype-stratified analysis was not performed for *A. baumannii* because genotype data were not available in ATLAS.

### Cross-resistance profile

**Fig 3:**
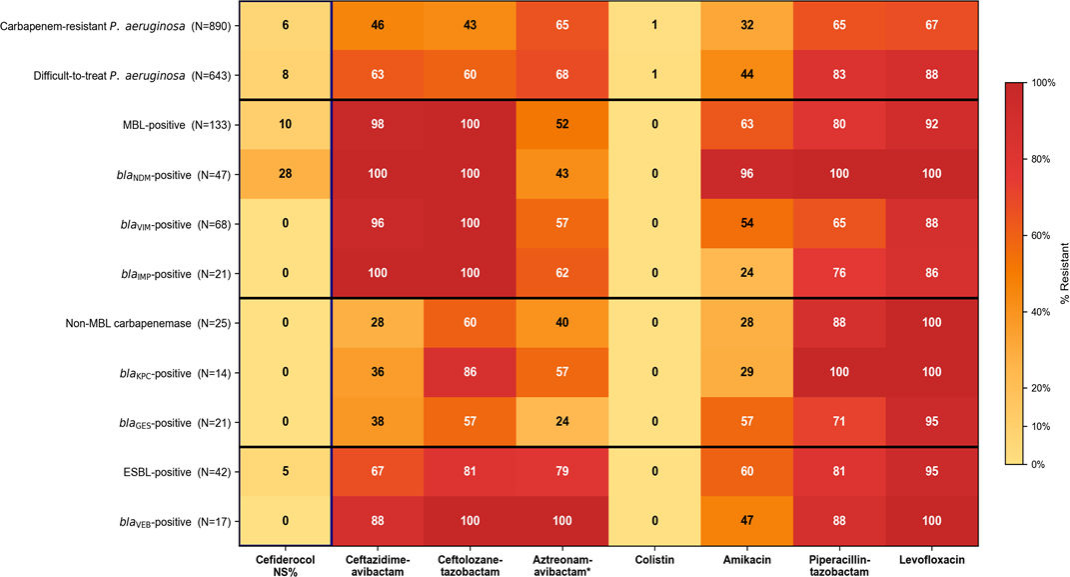
Heatmap of cefiderocol nonsusceptibility and comparator resistance by β-lactamase genotype/phenotype in *P. aeruginosa*. The first two rows summarize overall CRPA and DTRPA populations; the remaining rows are grouped by Ambler class: Class B MBL (MBL-positive umbrella plus *bla*_NDM_, *bla*_VIM_, and *bla*_IMP_), Class A serine carbapenemase (non-MBL carbapenemase plus *bla*_KPC_ and *bla*_GES_), and Class A ESBL (ESBL-positive plus *bla*_VEB_). For colistin, values represent % CLSI-resistant because CLSI 2024 has not defined a susceptible category for *P. aeruginosa* or *A. baumannii*. Aztreonam-avibactam breakpoint caveats are detailed in the figure footnote.

Although the majority of CRPA remained susceptible to ceftolozane-tazobactam and ceftazidime-avibactam, co-resistance was near-universal within the 54 cefiderocol-nonsusceptible CRPA: ceftazidime-avibactam 98.1%, ceftolozane-tazobactam 96.3%, amikacin 92.6%, piperacillin-tazobactam 94.4%, and levofloxacin 96.3%, with colistin resistance of only 3.7% (Table S2). Among 122 cefiderocol-nonsusceptible CRAB, amikacin resistance was 92.6%, gentamicin 97.5%, and levofloxacin 95.1%; colistin resistance was 0.8%. The heatmap (Fig. 3) showed *bla*_VIM_- and *bla*_IMP_-positive CRPA had 0% cefiderocol nonsusceptibility but 96–100% resistance to ceftazidime-avibactam and ceftolozane-tazobactam, highlighting cefiderocol as their sole effective β-lactam.

Aztreonam-avibactam MIC data were available for all CRPA isolates (Table 3). Among CRPA overall, 34.8% (310/890) had aztreonam-avibactam MICs ≤ 8 mg/L; descriptive activity was highest among *bla*_NDM_-positive isolates (57.4%, 27/47). The genotype-stratified resistance profile, including aztreonam-avibactam, is shown in Fig. 3. For CRAB, aztreonam-avibactam showed minimal activity (1.5%, MIC ≤ 8 mg/L).

**TABLE 3:** *In vitro* activity of salvage antimicrobial agents against cefiderocol-nonsusceptible and carbapenem-resistant *Pseudomonas aeruginosa* (CRPA) and carbapenem-resistant *Acinetobacter baumannii* (CRAB), ATLAS 2024, stratified by resistance group

Group	*N*	ATM-AVI ≤ 8 mg/L^*a*^	Colistin non-R^*b*^	Amikacin-S^*c*^	AmpSul-S^*c*^
*Pseudomonas aeruginosa*					
Carbapenem-resistant	890	310 (34.8)	884 (99.3)	550 (61.8)	NA^*d*^
Difficult-to-treat	643	207 (32.2)	637 (99.1)	314 (48.8)	NA
MBL-positive	133	64 (48.1)	133 (100.0)	35 (26.3)	NA
*bla*_NDM_-positive	47	27 (57.4)	47 (100.0)	1 (2.1)	NA
*bla*_VIM_-positive	68	29 (42.6)	68 (100.0)	19 (27.9)	NA
*bla*_IMP_-positive	21	8 (38.1)	21 (100.0)	15 (71.4)	NA
*Acinetobacter baumannii*					
Carbapenem-resistant	1,063	16 (1.5)	1,041 (97.9)	133 (12.5)	67 (6.3)
Difficult-to-treat	1,042	15 (1.4)	1,020 (97.9)	117 (11.2)	65 (6.2)

^*a*^ ATM-AVI ≤ 8 mg/L applies the FDA provisional breakpoint for Enterobacterales and is presented as a descriptive proportion only; the value likely represents an upper-bound estimate because the constant *in vitro* avibactam concentration may exceed trough concentrations achieved during clinical dosing.

^*b*^ Colistin is reported as % non-resistant (CLSI S + I) because CLSI 2024 defines no susceptible category for colistin against *P. aeruginosa *or *A. baumannii* (only intermediate and resistant).

^*c*^ Amikacin and AmpSul (ampicillin-sulbactam) susceptibility were interpreted using CLSI 2024 (M100, 34th edition) breakpoints.

^*d*^ NA, not applicable.

## DISCUSSION

This study provides the first genotype-stratified analysis of cefiderocol activity against CRPA and CRAB using the 2024 ATLAS data set. Three novel findings emerged: (i) non-carbapenemase mechanisms account for 76% of cefiderocol nonsusceptibility in CRPA; (ii) *bla*_NDM_ is the only MBL gene associated with nonsusceptibility, while *bla*_VIM_ and *bla*_IMP_ producers remain uniformly susceptible; and (iii) CRAB has significantly higher nonsusceptibility than CRPA (OR = 2.01, *P* < 0.001).

The CRPA nonsusceptibility rate (6.1%, CLSI) in this study exceeds those from earlier SIDERO-WT (0.1–0.2% for all *P. aeruginosa*) (11) and SENTRY (0.4%) (12) surveys. Updated SENTRY (2020–2023) data reported 98.1% cefiderocol susceptibility among DTR *P. aeruginosa* (CLSI), with 97.3% susceptibility in the carbapenemase-carrying subset (13). The 5-year SENTRY cumulative analysis (2020–2024) further showed sustained susceptibility of 95.3–100% among carbapenem-nonsusceptible *P. aeruginosa* and 87.0–97.7% among carbapenem-nonsusceptible *A. baumannii* complex across the United States and Europe (14). Our higher CRPA nonsusceptibility of 6.1% (CLSI) likely reflects broader geographic representation, including South Asia, where *bla*_NDM_-producing CRPA is endemic. A recent meta-analysis reported 3.5% for CRPA using EUCAST breakpoints (15); direct comparison is limited because EUCAST defines susceptibility at ≤2 mg/L (vs CLSI ≤ 4 mg/L), and our own EUCAST-based reanalysis yielded a higher CRPA nonsusceptibility rate (Table 1 footnote). For CRAB, the nonsusceptibility rate (11.5%) was comparable to the meta-analysis estimate (13.2%, using EUCAST PK/PD breakpoints extrapolated to *A. baumannii*) (15). The CRPA-lower, CRAB-higher nonsusceptibility pattern observed in our data set is consistent with a recent meta-analysis (CRPA 3.5% vs CRAB 13.2% under EUCAST) (15) and with European SENTRY data showing 93–100% cefiderocol susceptibility among CRAB across five countries (17). The higher rates likely reflect restriction to carbapenem-resistant isolates, broader geographic representation, including South Asia, and the 2024 collection period. In Taiwan, 100% cefiderocol susceptibility was reported among carbapenem-nonsusceptible *P. aeruginosa* (2018–2020) with MIC_50_/MIC_90_ of 0.25/1 mg/L (18), lower than our CRPA data (0.5/4 mg/L), which may reflect the inclusion of *bla*_NDM_-producing isolates from South Asia in the current study.

Among 54 cefiderocol-nonsusceptible CRPA, 41 (76%) lacked detectable carbapenemase genes, indicating that non-enzymatic mechanisms predominate at the population level. This complements *in vitro* data focused on MBL hydrolysis (5, 19). In a German study of 108 CRPA isolates, no correlation was found between carbapenemase carriage and cefiderocol resistance; instead, TonB-dependent receptor mutations were the primary determinants (20). Multiple non-enzymatic mechanisms have been described in *P. aeruginosa*: siderophore receptor mutations (*piuA*, *pirA*, *piuD*, and *piuC*) (21), AmpC Omega-loop mutations, efflux pump alterations in MexAB-OprM and MexCD-OprJ (22), and PBP3 target modifications (23). Some of these mechanisms confer collateral susceptibility to carbapenems (22), which may explain why non-carbapenemase CRPA isolates can simultaneously exhibit cefiderocol nonsusceptibility through overlapping pathways.

Among MBL-positive CRPA, nonsusceptibility was exclusively driven by *bla*_NDM_ producers (27.7%, 13/47), while *bla*_VIM_ (*n* = 68) and *bla*_IMP_ (*n* = 21) producers were 100% susceptible. *bla*_NDM_-encoded enzymes hydrolyze cefiderocol with catalytic efficiency approximately 100-fold higher than *bla*_VIM_ or *bla*_IMP_ products, owing to rapid dissociation of a reversible enzyme-product adduct (24). Cefiderocol hydrolysis by *bla*_IMP_- and *bla*_VIM_-encoded enzymes is 260-fold less efficient than meropenem hydrolysis, but only 3-fold less efficient for *bla*_NDM_ (19). These kinetics directly explain our MIC distributions (Fig. 1B through D). SIDERO-WT confirmed 100% susceptibility among MBL-producing *P. aeruginosa* (25), and SENTRY showed 97.3% susceptibility among carbapenemase-carrying DTR *P. aeruginosa* (13), reinforcing that MBL carriage alone does not predict nonsusceptibility. The emergence of *bla*_NDM_-producing strains replacing *bla*_KPC_-producing strains after ceftazidime-avibactam introduction, as documented in Taiwan (26), adds urgency to this distinction.

*bla*_VIM_- and *bla*_IMP_-producing *P. aeruginosa* were 96–100% resistant to ceftazidime-avibactam and ceftolozane-tazobactam, while maintaining 100% susceptibility to cefiderocol (Fig. 3). This positions cefiderocol as the only effective β-lactam for these genotypes, consistent with the IDSA 2024 guidance (4). Meta-analyses have demonstrated lower all-cause mortality with cefiderocol compared with colistin-based regimens (RR = 0.71, 95% CI = 0.54–0.92) (27), and Asian guidelines also support cefiderocol use in this setting (9, 10). However, no randomized trial has specifically compared cefiderocol with colistin for MBL-producing *P. aeruginosa* infections. Cefepime-taniborbactam, which is not currently FDA-approved (a Complete Response Letter was issued in February 2024), may eventually provide additional coverage for MBL-producing *P. aeruginosa*, but clinical data for non-urinary indications remain limited.

The complementary activity profiles of cefiderocol and aztreonam-avibactam across MBL subtypes carry therapeutic implications. *Bla*_NDM_-producing CRPA represented the most constrained subgroup: cefiderocol nonsusceptibility was 27.7%, and amikacin susceptibility was only 2.1%. In contrast, aztreonam-avibactam showed the highest descriptive activity in this subgroup (57.4% MIC ≤ 8 mg/L), consistent with the inability of *bla*_NDM_ to hydrolyze monobactams and avibactam-mediated inhibition of co-carried ESBL and chromosomal AmpC (28, 29). Among *bla*_VIM_- and *bla*_IMP_-positive isolates, cefiderocol retained 100% susceptibility while aztreonam-avibactam activity was lower (42.6% and 38.1% MIC ≤ 8 mg/L, respectively), confirming their non-interchangeable roles. These data position aztreonam-avibactam as a rational partner or alternative for cefiderocol-nonsusceptible *bla*_NDM_-producing CRPA, pending species-specific breakpoint establishment.

The cefiderocol nonsusceptibility rate was significantly higher in CRAB (11.5%) than in CRPA (6.1%). The results are consistent with meta-analysis data showing that cefiderocol nonsusceptibility is substantially higher in CRAB than in CRPA (13.2% vs 3.5%) (15). Several mechanistic factors may contribute. The iron transport system in *A. baumannii* is less redundant than in *P. aeruginosa; pirA* mutations alone can reduce cefiderocol susceptibility in *A. baumannii* (30), whereas *P. aeruginosa* possesses multiple compensatory siderophore receptors (21). In addition, *A. baumannii* frequently co-carries OXA-type carbapenemases that provide additional enzymatic hydrolysis; SIDERO-WT showed that only 60% of MBL-producing *A. baumannii* were cefiderocol-susceptible, compared with 100% of MBL-producing *P. aeruginosa* (25). Transmission of cefiderocol-nonsusceptible CRAB among cefiderocol-naive patients has also been documented (31).

Despite the higher nonsusceptibility rate in CRAB, cefiderocol retains an essential clinical role in this setting. In the 2024 ATLAS data set, alternative β-lactam options were substantially worse: aztreonam-avibactam had activity against only 1.5% of CRAB at MIC ≤ 8 mg/L, and standard-dose ampicillin-sulbactam against 6.3% (Table 3). Colistin remained the only comparator with broad retained activity (97.9% non-resistant, a value that reflects absence of resistance rather than confirmed susceptibility because CLSI defines no susceptible category for colistin), but nephrotoxicity rates of up to 36.2% limit its durability (32), and a meta-analysis reported lower all-cause mortality with cefiderocol than colistin-based regimens for CRAB bloodstream infection (RR = 0.71, 95% CI = 0.54–0.92), although no significant survival advantage was observed in the ventilator-associated pneumonia subgroup (27). The PROVE observational study further demonstrated that earlier initiation of cefiderocol was associated with clinical cure rates of 73.7% (empirical use) vs 54.3% (salvage use), reinforcing the value of susceptibility-guided early use (33). One potential contributing factor to the clinical attenuation of cefiderocol in CRAB is heteroresistance in *A. baumannii*, which can be induced upon exposure to human pleural fluid through altered iron-uptake regulation and β-lactamase expression, potentially widening the gap between *in vitro* susceptibility and clinical response (34). Although heteroresistance to cefiderocol has been reported in CRAB, available real-world data do not support it as the dominant mechanism of treatment failure (35). Cefiderocol shows consistent clinical effectiveness in CRAB and MDR gram-negative infections, particularly in bloodstream infections. Treatment outcomes are more likely driven by infection site, illness severity, and pharmacokinetic factors in critically ill patients (36, 37). In the Indian setting, where our CRPA and CRAB nonsusceptibility rates were highest (43.4% and 48.3%), recent data similarly confirmed that cefiderocol retains activity against a meaningful proportion of CRAB despite high endemic carbapenemase prevalence (38). These findings support the continued positioning of cefiderocol within CRAB treatment algorithms alongside high-dose sulbactam-containing combinations.

Among 54 cefiderocol-nonsusceptible CRPA, concurrent resistance rates to ceftazidime-avibactam (98.1%), ceftolozane-tazobactam (96.3%), and amikacin (92.6%) confirmed near pan-resistance among classical comparators; only colistin (resistance 3.7%) and aztreonam-avibactam (descriptive activity in the MBL subgroup; Fig. 3) retained *in vitro* activity. Cefiderocol-nonsusceptible CRAB showed similar co-resistance to amikacin (92.6%), gentamicin (97.5%), and levofloxacin (95.1%), with colistin resistance of only 0.8%. Once cefiderocol susceptibility is lost, treatment options are essentially limited to colistin, an agent associated with nephrotoxicity rates of up to 36.2% (32). Sulbactam-durlobactam and minocycline, which are increasingly relevant for CRAB salvage therapy, were not available in the ATLAS 2024 panel and could not be characterized. Aztreonam-avibactam data should be interpreted with the caveat that ATLAS employs a surrogate method (aztreonam with fixed 4 mg/L avibactam) and that no species-specific clinical breakpoint exists for *P. aeruginosa*. These findings underscore the importance of antimicrobial stewardship to preserve cefiderocol activity (39) and active surveillance to monitor emerging resistance.

Geographically, cefiderocol nonsusceptibility in CRPA was concentrated in South Asia, with India having the highest rate (43.4%), followed by Nigeria (18.8%) and Greece (17.1%). This pattern mirrors the epidemiology of *bla*_NDM_-producing *P. aeruginosa* (2). CRAB nonsusceptibility was more widely dispersed: Ukraine (50.0%), India (48.3%), South Africa (33.3%), and Turkey (14.6%), consistent with the global dissemination of OXA-23-carrying CRAB clones (40). Recent data from India, where carbapenemase prevalence is high, reported substantial cefiderocol nonsusceptibility among carbapenem-resistant *A. baumannii*, predominantly linked to endemic NDM-producing clones (38). In contrast, European SENTRY data (2020–2023) from Italy, France, Germany, Spain, and the United Kingdom demonstrated 93–96% cefiderocol susceptibility against *A. baumannii* complex despite varying carbapenem-resistance prevalence, reflecting the heterogeneous regional resistance landscape (17). Asia had the highest nonsusceptibility for both CRPA (14.8%) and CRAB (21.3%), while Latin America had rates approaching zero. The contrast between CRPA (geographically clustered) and CRAB (geographically dispersed) has implications for empiric therapy and region-specific surveillance.

The documentation of *bla*_NDM_ co-carriage with *bla*_OXA_ on mobile genetic elements in Taiwan (41) highlights the potential for *bla*_NDM_ dissemination to previously low-prevalence regions. This study extends the evidence base beyond SIDERO-WT (11) and SENTRY (12, 13) in several ways. First, the 2024 data set captures a more contemporary resistance landscape. Second, the analysis focuses specifically on carbapenem-resistant and DTR subpopulations, providing clinically actionable data for the most challenging infections. Third, the genotype-stratified analysis revealing *bla*_NDM_ as the sole MBL gene affecting cefiderocol activity, with direct visualization of MIC distributions by MBL subtype (Fig. 1), has not been previously reported in global surveillance. Fourth, the cross-resistance heatmap (Fig. 3) demonstrates that cefiderocol and colistin are the only agents retaining activity across all genotype groups.

Several limitations should be acknowledged. First, the ATLAS program uses convenience sampling rather than population-based surveillance, potentially introducing selection bias. Second, this cross-sectional, single-year analysis (2024) precludes temporal trend assessment. Third, ATLAS provides β-lactamase genotype data for *P. aeruginosa* but not for *A. baumannii*; consequently, the molecular basis of CRAB nonsusceptibility could not be characterized. Fourth, the *P. aeruginosa* genotype data are limited to Class A and B β-lactamases; non-enzymatic mechanisms (siderophore receptor mutations, efflux pump overexpression, and PBP3 modifications) require whole-genome sequencing for definitive characterization. Fifth, molecular screening was performed only on isolates meeting predefined MIC triage criteria, not on the entire CRPA collection; therefore, genotype prevalence estimates may not represent the overall population. Sixth, CLSI breakpoints were used; application of EUCAST breakpoints would yield higher nonsusceptibility rates (15), and EUCAST has issued a technical warning regarding commercially available cefiderocol susceptibility tests (42). Seventh, the DTR classification depends on the availability of MIC results for all qualifying agents; although all agents were routinely tested, any variation in panel completeness could affect the DTR denominator. Eighth, geographic representation was uneven; the United States was not included in the 2024 data set. Ninth, aztreonam-avibactam susceptibility was determined using a surrogate method with fixed 4 mg/L avibactam; the constant *in vitro* avibactam concentration may exceed trough concentrations achieved during clinical dosing, and the reported MIC ≤ 8 mg/L proportions likely represent upper-bound estimates. Tenth, other agents increasingly relevant to CRAB salvage therapy, including sulbactam-durlobactam, minocycline, and cefepime-taniborbactam, were not part of the ATLAS 2024 panel and could not be characterized. Eleventh, clinical outcome data were not captured. Twelfth, cefiderocol-intermediate and cefiderocol-resistant isolates were not retested; given the recognized variability of cefiderocol broth microdilution MIC testing, single-determination results may be subject to misclassification. Thirteenth, isolates were contributed by a limited number of participating laboratories within each country and may not be representative of all isolates or geographic areas within that country; reported rates may over- or under-represent true national antimicrobial susceptibility.

In conclusion, cefiderocol retained good *in vitro* activity against CRPA (93.9% susceptible) and CRAB (88.5% susceptible) in the 2024 ATLAS data set. Among CRPA, non-carbapenemase mechanisms accounted for 76% of cefiderocol-nonsusceptible isolates, and *bla*_NDM_ was the only MBL gene associated with nonsusceptibility. *bla*_VIM_- and *bla*_IMP_-producing isolates were uniformly susceptible, positioning cefiderocol as their sole effective β-lactam. CRAB exhibited significantly higher nonsusceptibility, likely driven by species-specific resistance determinants requiring further characterization. These findings support the continued role of cefiderocol in treating DTR *P. aeruginosa* and *A. baumannii* infections and underscore the need for genotype-aware susceptibility testing and global surveillance.

## MATERIALS AND METHODS

### Collection and identification of isolates

This study includes clinically relevant causative isolates of *P. aeruginosa* and *A. baumannii* collected in 2024 through the ATLAS global surveillance program, from hospitalized patients with bloodstream, intra-abdominal, lower respiratory tract, skin and soft tissue, or urinary tract infections (16). To avoid duplicate sampling, only the first isolate of a given species from each patient was included during the collection period. Isolates with unknown patient age were excluded from age-stratified analyses. Species identification was confirmed at the central laboratory (International Health Management Associates, Schaumburg, IL, USA) using matrix-assisted laser desorption ionization time-of-flight mass spectrometry (MALDI-TOF MS; Bruker Daltonics, Billerica, MA, USA).

### Antimicrobial susceptibility testing

Following identification, isolates were tested at the International Health Management Associates, Inc. central laboratory using custom 96-well broth microdilution panels. Cefiderocol MICs were determined using iron-depleted cation-adjusted Mueller-Hinton broth, as stipulated by the CLSI reference method for cefiderocol susceptibility testing (43). MICs of comparator agents were determined using standard cation-adjusted Mueller-Hinton broth. Comparator agents included ceftazidime-avibactam and ceftolozane-tazobactam for *P. aeruginosa*; amikacin, piperacillin-tazobactam, levofloxacin, and colistin for both species; and gentamicin and ampicillin-sulbactam additionally for *A. baumannii*.

All MICs were interpreted using 2024 CLSI (M100, 34th edition) breakpoints (43). For cefiderocol, CLSI breakpoints define an MIC of ≤4 mg/L as susceptible, 8 mg/L as intermediate, and ≥16 mg/L as resistant. Nonsusceptibility was defined as an MIC ≥8 mg/L (CLSI intermediate + resistant) throughout this study. For *P. aeruginosa*, EUCAST (version 14.0, 2024) cefiderocol breakpoints (susceptible ≤2 mg/L, resistant >2 mg/L) were also applied for comparison (44). EUCAST has not established clinical breakpoints for cefiderocol against *A. baumannii* (designated as “insufficient evidence”); therefore, EUCAST interpretations were not applied to *A. baumannii* isolates. CLSI was applied as the primary interpretive system for both species; for *P. aeruginosa*, EUCAST results are reported alongside CLSI for comparison. Because the EUCAST susceptible breakpoint for cefiderocol (≤2 mg/L) is lower than the CLSI breakpoint (≤4 mg/L), the two systems produce different nonsusceptibility estimates for *P. aeruginosa*, with the EUCAST estimate being the higher of the two (Table 1). For colistin, CLSI 2024 defines only intermediate and resistant categories for *P. aeruginosa* and *A. baumannii* and does not provide a susceptible category, whereas EUCAST defines colistin breakpoints (susceptible ≤2 mg/L, resistant >2 mg/L) for both species. To avoid implying a susceptibility category that CLSI does not provide, colistin results are reported here as the proportion non-resistant under CLSI rather than as a percenage susceptible.

Aztreonam-avibactam minimum inhibitory concentrations were determined by a surrogate method using aztreonam in combination with a fixed concentration of avibactam (4 mg/L). Because neither CLSI nor EUCAST has established clinical breakpoints for aztreonam-avibactam against *P. aeruginosa* or *A. baumannii*, the FDA provisional breakpoint for Enterobacterales (susceptible ≤8 mg/L) was applied for descriptive purposes only and is reported as the proportion at ≤8 mg/L (Table 3).

### Resistance definitions

CRPA was defined as meropenem-resistant per CLSI breakpoints (MIC ≥ 8 mg/L) (43). CRAB was defined as meropenem-resistant per CLSI breakpoints (MIC ≥ 8 mg/L). DTRPA was defined as nonsusceptible to all of the following: piperacillin-tazobactam, ceftazidime, cefepime, aztreonam, meropenem, imipenem-cilastatin, ciprofloxacin, and levofloxacin, based on the classification by Kadri et al. (3). DTRAB was defined as nonsusceptible to all of: imipenem, meropenem, ampicillin-sulbactam, ciprofloxacin, and levofloxacin (3). All agents used for DTR classification were routinely tested on the ATLAS broth microdilution panel; isolates with missing MIC results for any qualifying agent were excluded from DTR classification. DTR was identified using the ATLAS difficult-to-treat resistance phenotype, which applies the definition of Kadri et al. (3) to the entire species population independently of the carbapenem-resistance definition; consequently, the CR and DTR populations overlap but are not nested.

### β-Lactamase gene screening

Under the ATLAS workflow, *P. aeruginosa* isolates meeting predefined MIC triage criteria (meropenem MIC > 1 mg/L, ceftazidime-avibactam MIC > 8 mg/L, or aztreonam-avibactam MIC > 8 mg/L) were submitted for molecular testing. Multiplex polymerase chain reaction at the reference laboratory targeted MBLs (*bla*_NDM_, *bla*_IMP_, and *bla*_VIM_), serine carbapenemases (*bla*_KPC_ and *bla*_GES_), and extended-spectrum β-lactamases (ESBLs; *bla*_CTX-M_, *bla*_PER_, *bla*_VEB_, and *bla*_TEM_), followed by amplification and sequencing of full-length genes (16). Isolates were classified as MBL-positive when harboring at least one of *bla*_NDM_, *bla*_VIM_, or *bla*_IMP_. Carbapenemase-positive/MBL-negative isolates were those carrying *bla*_KPC_ or *bla*_GES_ without co-carriage of any MBL gene. Non-carbapenemase-producing CRPA were defined by subtraction (CRPA total minus MBL-positive minus carbapenemase-positive/MBL-negative). Because individual isolates may carry more than one β-lactamase gene, gene categories are not mutually exclusive. Genotype data were available only for *P. aeruginosa* and limited to Class A and B β-lactamases. No genotype data were available for *A. baumannii* in the ATLAS program.

### Cross-resistance analysis

Cross-resistance between cefiderocol and each comparator agent was assessed using joint susceptibility distributions, in which cefiderocol and comparator susceptibility categories were cross-tabulated. For *P. aeruginosa*, cross-resistance was further stratified by β-lactamase genotype group to generate a resistance profile heatmap (Fig. 3). Cefiderocol values in the heatmap represent nonsusceptibility rates (CLSI I + R); comparator values represent resistance rates (CLSI R only), because colistin lacks a CLSI susceptible category for *P. aeruginosa* (only intermediate and resistant are defined).

### Statistical analysis

Between-group differences in cefiderocol nonsusceptibility rates were expressed as odds ratios (ORs) with 95% confidence intervals (CIs) and tested using two-sided Fisher’s exact tests when any expected cell count was <5, or chi-squared tests with Yates’ correction otherwise (*P* < 0.05). Comparisons were performed across species (CRPA vs CRAB), continent, specimen source, ward type, age group, and genotype. Countries contributing fewer than 10 isolates were excluded from geographic analyses. Data were extracted from the ATLAS online platform (http://www.atlas-surveillance.com).

## Data Availability

The ATLAS surveillance data analyzed in this study were provided by Pfizer Inc. (the sponsor of the ATLAS program). These data are not publicly available due to data sharing restrictions associated with the surveillance program license; however, aggregated summary data supporting the findings of this study are available from the corresponding author upon reasonable request and with permission of Pfizer Inc.

## SUPPLEMENTAL MATERIAL

Supplemental tables aac.00634-26-s0001.docx Tables S1 and S2.

ASM does not own the copyrights to Supplemental Material that may be linked to, or accessed through, an article. The authors have granted ASM a non-exclusive, world-wide license to publish the Supplemental Material files. Please contact the corresponding author directly for reuse.
