# Supplementary material for: Cefiderocol nonsusceptibility among carbapenem-resistant Pseudomonas aeruginosa and Acinetobacter baumannii: data from the 2024 Antimicrobial Testing Leadership and Surveillance (ATLAS) program
Source: Antimicrob Agents Chemother. 2026 Jul 13;70(8):e00634-26. doi: 10.1128/aac.00634-26 (PMC13436418; doi:10.1128/aac.00634-26)
Supplement: Supplementary file 1 [file aac.00634-26-s0001.docx]

**Supplementary TABLE S1.** β-Lactamase gene distribution among cefiderocol-nonsusceptible CRPA, by MBL status

| **Gene** | **Total FDC-NS (n = 54)** | **MBL (+) (n = 13)** | **MBL (−) (n = 41)** |
| --- | --- | --- | --- |
| **Carbapenemase status** |  |  |  |
| MBL-positive | 13 (24.1%) | 13 (100%) | — |
| Carbapenemase(+)/MBL(−) | 0 (0%) | — | 0 (0%) |
| Non-carbapenemase | 41 (75.9%) | — | 41 (100%) |
| **Class B (MBL)** |  |  |  |
| *bla*_NDM_ | 13 (24.1%) | 13 (100%) | — |
|  |  |  |  |
| *bla*_VIM_ | 0 (0%) | 0 (0%) | — |
| *bla*_IMP_ | 0 (0%) | 0 (0%) | — |
| **Class A (Serine carbapenemases)** |  |  |  |
| *bla*_KPC_ | 0 (0%) | 0 (0%) | 0 (0%) |
| *bla*_GES_ | 0 (0%) | 0 (0%) | 0 (0%) |
| **Class A (ESBL)** |  |  |  |
| *bla*_CTX-M_ | 0 (0%) | 0 (0%) | 0 (0%) |
| *bla*_PER_ | 0 (0%) | 0 (0%) | 0 (0%) |
| *bla*_VEB_ | 0 (0%) | 0 (0%) | 0 (0%) |
| *bla*_TEM_ | 0 (0%) | 0 (0%) | 0 (0%) |

FDC-NS, cefiderocol-nonsusceptible (CLSI I + R, MIC ≥ 8 mg/L).

Non-carbapenemase = CRPA total (n = 890) − MBL(+) − Carbapenemase(+)/MBL(−); defined by subtraction. Three isolates co-harbored more than one MBL gene.

**Supplementary TABLE S2.** Cross-resistance between cefiderocol and comparator agents among CRPA and CRAB (ATLAS 2024)

| **Comparator** | **FDC-S/ Comp-S** | **FDC-S/ Comp-R** | **FDC-NS/ Comp-S** | **FDC-NS/ Comp-R** | **FDC-NS total** | **Comp-R among FDC-NS (%)** |
| --- | --- | --- | --- | --- | --- | --- |
| ***P. aeruginosa* (CRPA, N=890)** |  |  |  |  |  |  |
| Ceftazidime-avibactam | 479 | 357 | 1 | 53 | 54 | 98.1 |
| Ceftolozane-tazobactam | 444 | 334 | 2 | 52 | 54 | 96.3 |
| Colistin | 0 | 4 | 0 | 2 | 54 | 3.7 |
| Amikacin | 547 | 233 | 3 | 50 | 54 | 92.6 |
| Piperacillin-tazobactam | 191 | 530 | 1 | 51 | 54 | 94.4 |
| Levofloxacin | 174 | 545 | 1 | 52 | 54 | 96.3 |
| ***A. baumannii* (CRAB, N=1063)** |  |  |  |  |  |  |
| Colistin | 0 | 21 | 0 | 1 | 122 | 0.8 |
| Amikacin | 126 | 799 | 7 | 113 | 122 | 92.6 |
| Gentamicin | 96 | 806 | 2 | 119 | 122 | 97.5 |
| Ampicillin-sulbactam | 66 | 659 | 1 | 78 | 122 | 63.9 |
| Levofloxacin | 29 | 847 | 0 | 116 | 122 | 95.1 |

FDC-S, MIC ≤ 4; FDC-NS, MIC ≥ 8 (CLSI I + R). Comp-S/R by CLSI breakpoints. Intermediate categories omitted.
